# The Role of CT Perfusion in Differentiating Benign Versus Malignant Focal Pulmonary Lesions

**DOI:** 10.7759/cureus.63618

**Published:** 2024-07-01

**Authors:** Kriti Kundu, Aman Kumar, Rajesh Malik, Radha Sarawagi, Alkesh Khurana, Jitendra Sharma, Abhinav C Bhagat, Ankur Patel

**Affiliations:** 1 Radiology, All India Institute of Medical Sciences, Bhopal, Bhopal, IND; 2 Pulmonary and Critical Care Medicine, All India Institute of Medical Sciences, Bhopal, Bhopal, IND

**Keywords:** benign lung lesions, malignant, pulmonary nodule characterization, lung cancer, differentiate malignant and benign lung lesions, focal lung lesions, ct perfusion

## Abstract

Background: Contrast-enhanced CT scan is the standard imaging for the characterization and evaluation of focal parenchymal lung lesions. It relies on morphology and enhancement patterns for the characterization of lung lesions. However, there is significant overlap among imaging features of various malignant and benign lesions. Hence, it is often necessary to obtain tissue diagnosis with invasive percutaneous or endoscopic-guided tissue sampling. It is often desirable to have non-invasive techniques that can differentiate malignant and benign lung lesions. CT perfusion is an emerging CT technology that allows functional assessment of tissue vascularity through various parameters and can help in differentiating benign and malignant focal lung lesions.

Objective: The purpose of this study was to assess the role of the CT perfusion technique in differentiating malignant and benign focal parenchymal lung lesions.

Materials and methods: In this prospective observational study, CT perfusion was performed on 41 patients with focal parenchymal lung lesions from December 2020 to June 2022. The four-dimensional range was planned to cover the entire craniocaudal extent of the lesion, followed by a volume perfusion CT (VPCT) of the lesion. A total of 27 dynamic datasets were acquired with a scan interval of 1.5 seconds and a total scan time of 42 seconds. CT perfusion parameters of blood flow (BF), blood volume (BV), and k-trans of the lesion were measured with mathematical algorithms available in the Syngo.via CT perfusion software (Siemens Healthcare, Erlangen, Germany).

Results: The median BV in benign lesions was found to be 5.5 mL/100 g, with an interquartile range of 3.3-6.9 and a p-value < 0.001. The median BV in malignant lesions was found to be 11.35 mL/100 g, with an interquartile range of 9.57-13.21 and a p-value ≤ 0.001. The median BF for benign lesions was 45.5 mL/100 g/min, with an interquartile range of 33.8-48.5 and a p-value ≤ 0.001. The median BF for malignant lesion was 61.77 mL/100 g/min, with an interquartile range of 33.8-48.5 and a p-value ≤ 0.001. The median k-trans in the case of benign lesions was found to be 4.2 mL/100 g/min, with an interquartile range of 3.13-6.8 and a p-value ≤ 0.001. The median k-trans in the case of the malignant lesion was found to be 12.05 mL/100g/min, with an interquartile range of 7.20-33.42 and a p-value < 0.001. Our study has also shown BV to have an accuracy of 92.68%, sensitivity of 93.3%, and specificity of 90.01%.

Conclusion: Our study has shown that CT perfusion values of BV, BF, and k-trans can be used to differentiate between benign and malignant focal lung parenchymal lesions. K-trans is the most sensitive parameter while BV and BF have greater accuracy and specificity.

## Introduction

Computed tomography perfusion (CTP) is an emerging technique that allows functional assessment of tissue vascularity through various parameters. With rapid technological advancements in multi-detector CT systems and the wide availability of commercial CTP software, the practical application of CT perfusion in routine imaging has broadened. CT perfusion is being utilized in both oncological and non-oncologic pathologies. In oncology, it is used for lesion characterization (differentiation between benign and malignant lesions), identification of occult malignancies, provision of prognostic information based on tumor vascularity, and monitoring therapeutic effects of the various treatment regimens, including chemoradiation and antiangiogenic drugs [1]. Non-oncologic applications include distinguishing between renal stenosis with and without preserved perfusion [2], observing hemodynamic changes in cirrhosis [3], recognizing areas of ischemia in acute pancreatitis [4], and differentiation of salvageable ischemic penumbra from unsalvageable core infarct in stroke, which helps identify patients most likely to benefit from thrombectomy or thrombolysis [5]. Despite improvements in therapeutic management, lung cancer stands among the leading causes of cancer-related death in the world and is expected to remain so in the future.

Nodules or masses discovered on chest X-ray or CT need further characterization, as benign or malignant, to initiate proper patient management. Contrast-enhanced CT relies on morphology and enhancement characteristics of lesions to differentiate malignant lesions from benign lesions. However, due to the overlapping morphology of various benign and malignant lesions, an image-guided biopsy is often required for confirmation. Image-guided core biopsy or fine needle aspirations have 95% to 100% accuracy in diagnosing malignant lesions and 91% accuracy in diagnosing benign lesions. However, limited expertise, invasiveness, and potential complications like pneumothorax limit their widespread application. Positron emission tomography-CT (PET-CT), though highly accurate, poses limitations of higher costs and availability, among imaging limitations of smaller (<10 mm) lesions, altered biodistribution of fluorodeoxyglucose (FDG) related to hyperglycemia or hyperinsulinemia, bone marrow activation, and motion artifacts [6,7]. Few studies have been attempted to evaluate perfusion parameters in benign and malignant lung lesions [6,8-10]. Most of them have shown perfusion parameters to be higher in malignant lesions as compared to benign ones. Even so, standardization of the perfusion values is not yet done in any study, and histological sampling remains the gold standard in subtyping.

## Materials and methods

Study population

A prospective observational study was conducted after getting approval from the Institutional Human Ethics Committee. Written informed consent was taken from all patients included in the study. A total of 41 patients who had undergone CT perfusion and image-guided biopsy were included in the study. Exclusion criteria included patients who had respiratory dysfunction, previous reactions to iodinated contrast media, pregnant females, patients who did not consent to be part of the study, patients who did not have tissue diagnosis, and patients whose histopathological results were inconclusive.

Imaging technique

CT Perfusion Acquisition

A non-enhanced chest CT of the lung was done to localize the pulmonary lesion with a 2 × 128-row dual-source CT scanner (Somatom Definition Flash, Siemens Healthcare, Erlangen, Germany). Subsequently, volume perfusion CT (VPCT) was done to cover the craniocaudal extent of the lesion with the acquisition of 27 dynamic datasets with a scan interval of 1.5 seconds and a total scan time of 42 seconds. During perfusion scanning, the patients were asked to withhold breathing during scanning and permitted shallow breathing at the end of scanning. A total of 50 ml of non-ionic iodinated contrast medium with an iodine concentration of 350 mg/ml (Omnipaque 350, GE HealthCare, Marlborough, MA) was injected at a flow rate of 6 mL/s in an antecubital vein, followed by a saline flush of 40 mL NaCl at 6.0 mL/s, which was started at a five-second delay. From the VPCT raw data, the axial images for perfusion analysis were reconstructed. All images were transferred to an external workstation for analysis.

**Figure 1:**
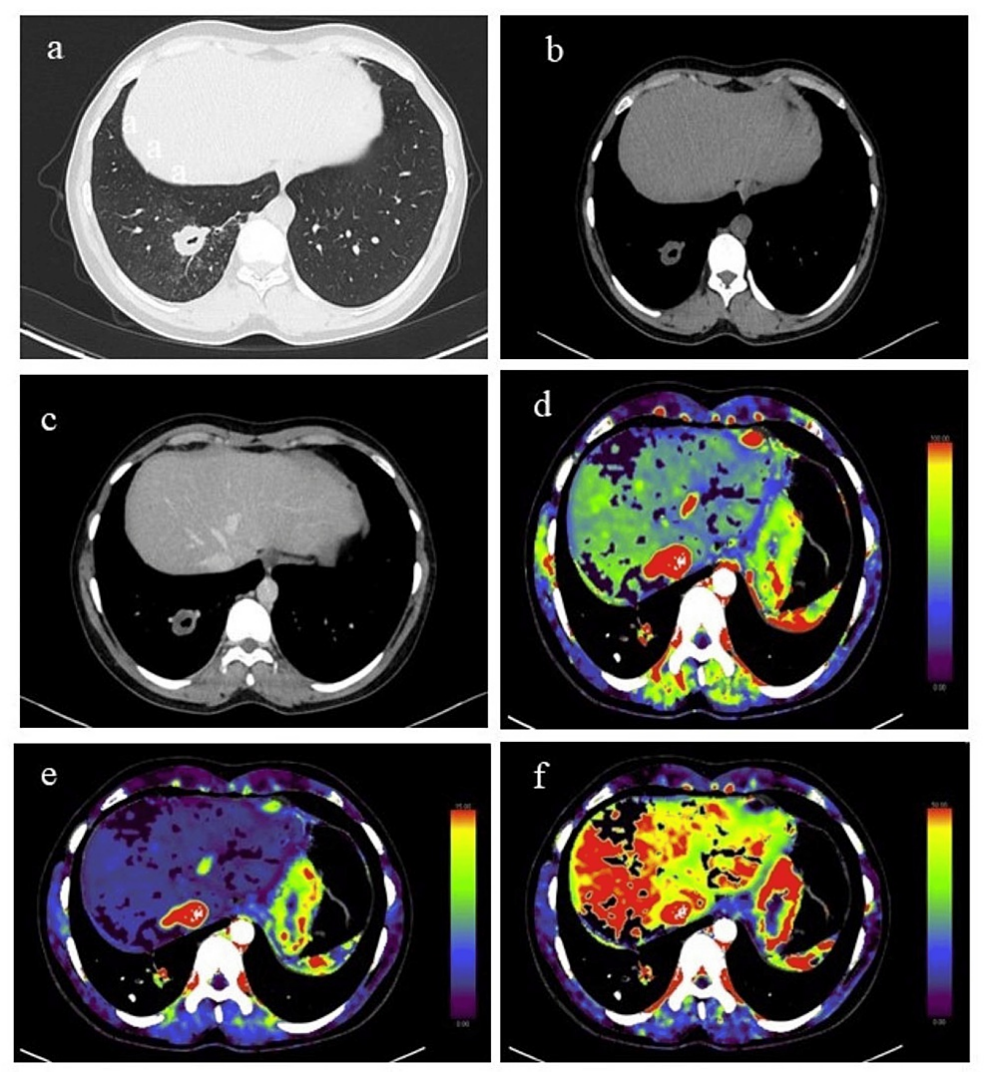
CT perfusion of a lung nodule. A 25-year-old male presented with a history of hemoptysis, fever, and shortness of breath. (b) Non-contrast (mediastinal window) CT showed a well-defined cavitatory lesion with smooth margins appreciated on the axial section lung window (a) in the posterobasal segment of the right lower lobe, which on post-contrast scan showed mild heterogeneous enhancement (c). Color-coded maps of CT perfusion: (d) blood flow (BF), (e) blood volume (BV), and (f) k-trans show areas of red and green indicating slightly high perfusion. The parameters were as follows: BV = 0.65, BF = 10.83, and k-trans = 3.31. On histopathology and microbiology correlation, the lesion turned out to be tubercular.

Quantitative data were evaluated with CT perfusion software (Syngo Volume Perfusion CT Body, Siemens Healthcare), which utilizes time attenuation curves (TAC) to assess tissue perfusion. An arterial input function was measured by placing the region of interest (ROI) in the thoracic aorta. Another ROI was placed in the pulmonary trunk for the subtraction of pulmonary vessels to avoid pulmonary contamination near the lesion. A manual ROI was drawn and defined in the transverse section of every slice around the lesion to cover the entire tumor volume. This was visually determined by the maximum intensity projections (MIP) image. The functional parameters obtained by CTP include the following: blood flow (BF; in mL/100 g/min) - flow rate through the vasculature in a tissue region; blood volume (BV; in mL/100 g) - the volume of flowing blood within a vasculature in a tissue region; and permeability (denoted as K-trans/flow extraction product in mL/100 g/min) - total flux from plasma to interstitial space. BF, BV, and k-trans of the lesion were calculated with mathematical algorithms available in the Syngo.via CT perfusion software (Siemens Healthcare) (Figure 1).

Histopathological and clinical correlation

The patients with lesions were followed up for their histopathological and clinical outcomes. Findings such as the lesion diagnosis and whether the lesion is benign or malignant were recorded.

## Results

Final histopathological diagnosis

A total of 41 patients with histopathological diagnoses were included for final analysis. Out of these 41 patients, 30 (73.1%) had malignant lesions and 11 (26.7%) had benign lesions. Out of 30 malignant lesions, 15 were adenocarcinomas, 12 were squamous cell carcinomas, and three were small cell carcinomas. Out of 11 benign lesions, eight were tubercular, one was fungal, one was mesenchymal neoplasm, and one was mesenchymal myxoid neoplasm.

Perfusion analysis

As our data had outliers, the median of perfusion parameters was calculated (Table 1).

**Table 1:** Comparison of perfusion parameters between benign and malignant lesions. BV: blood volume; BF: blood flow.

Perfusion parameters	Benign lesion (n = 11)	Malignant lesion (n = 30)	p-value
BV (mL/100 g)	5.5 (3.3-6.9)	11.35 (9.57-13.21)	<0.001
BF (mL/100 g/min)	45.5 (33.8-48.5)	61.77 (55.99-69.24)	<0.001
k-trans (mL/100 g/min)	4.2 (3.13-6.8)	12.05 (7.20-33.42)	<0.001

The median BV in the case of benign lesions was found to be 5.5 ml/100 g, with an interquartile range of 3.3-6.9 ml/100 g and a p-value ≤ 0.001. The median BV in the case of malignant lesions was found to be 11.35 ml/100 g, with an interquartile range of 9.57-13.21 ml/100 g and a p-value ≤ 0.001.

The median BF for benign lesion was 45.5 mL/100 g/min, with an interquartile range of 33.8-48.5 mL/100 g/min and a p-value ≤ 0.001. The median BF for malignant lesion was 61.77 mL/100 g/min, with an interquartile range of 55.99-69.24 mL/100 g/min and a p-value ≤ 0.001.

The median k-trans in the case of a benign lesion was found to be 4.2 mL/100 g/min, with an interquartile range of 3.13-6.8 mL/100 g/min and a p-value ≤ 0.001. The median k-trans in the case of a malignant lesion was found to be 12.05 mL/100 g/min, with an interquartile range of 7.20-33.42 and a p-value ≤ 0.001.

**Figure 2:**
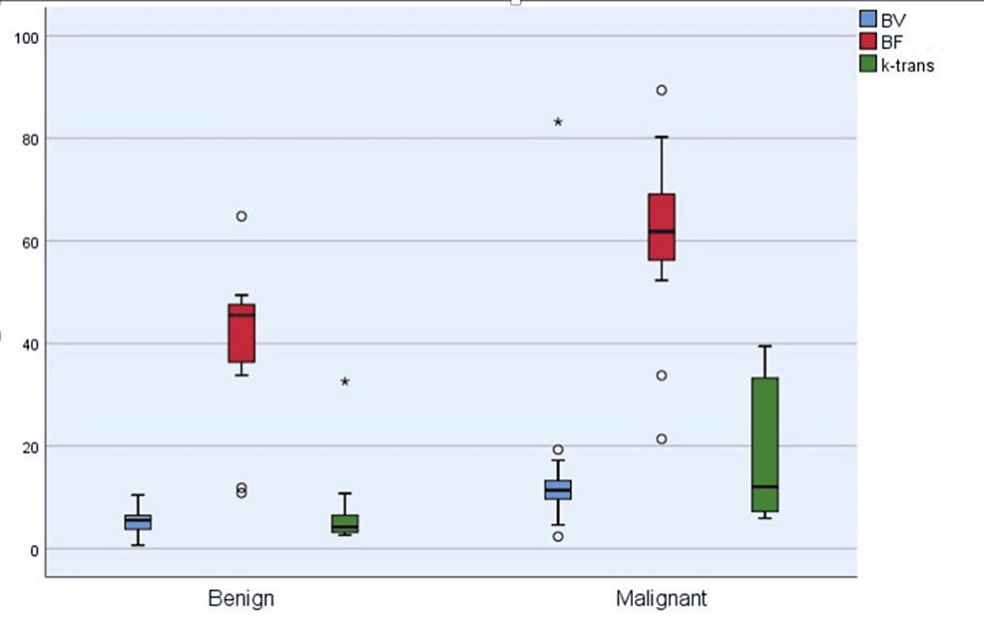
Box plot showing the comparison of perfusion parameters between benign and malignant lesions. BV: blood volume; BF: blood flow.

The box plot graphs were drawn comparing perfusion parameters of benign and malignant lesions. There was a significant difference in both groups (Figure 2).

**Figure 3:**
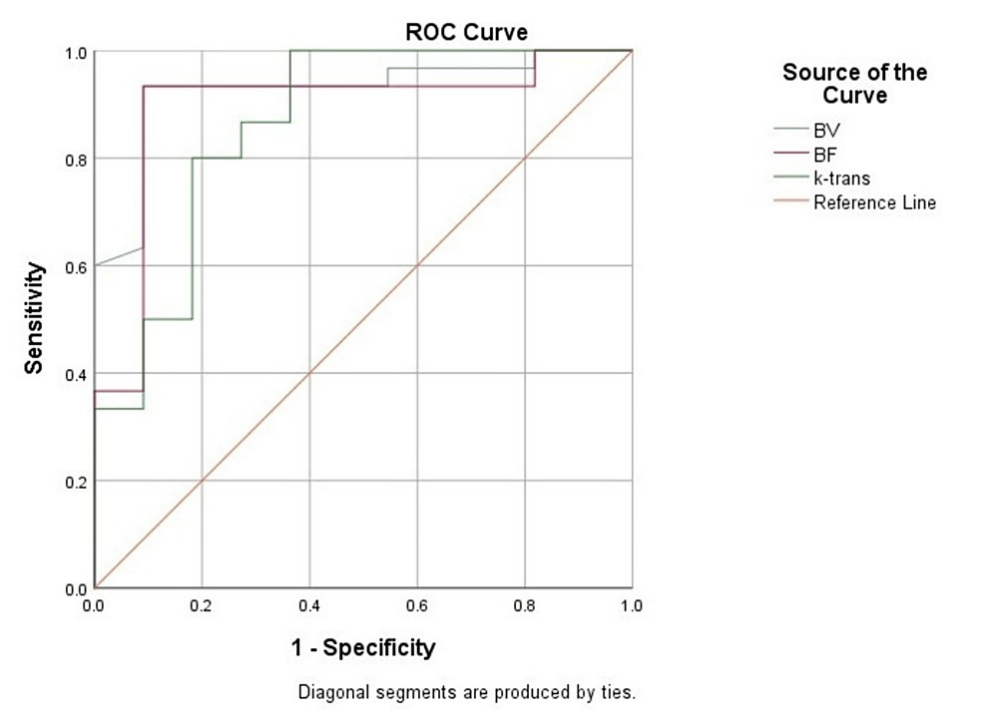
Receiver operating characteristic (ROC) curves of the three perfusion parameters for identifying malignant lesions. BV: blood volume; BF: blood flow.

The area under the receiver operating characteristic (ROC) curve for BV was 0.92, for BF was 0.89, and for k-trans was 0.86, thus demonstrating excellent diagnostic performance (Figure 3 and Table 2). The 95% confidence interval for BV was 0.83-1, for BF was 0.77-1, and for k-trans was 0.72-1. The Youden index for BV was 0.842, for BF was 0.84, and for k-trans was 0.636. All these values suggest that BV, BF, and k-trans can be used as parameters to differentiate between benign and malignant lesions. The sensitivity for BV was found to be 93.3%, for BF was 93.3%, and for k-trans was 100%. The specificity for BV was found to be 90.91%, for BF was 90.91%, and for k-trans was 63.64%. The positive predictive value (PPV) for BV was found to be 96.55%, for BF was 96.55%, and for k-trans was 88.24%. The negative predictive value (NPV) for BV was found to be 83.33%, for BF was 83.33%, and for k-trans was 100%. The accuracy for BV was found to be 92.68%, for BF was 92.68%, and for k-trans was 90.24%. The positive likelihood ratio (LR+) for BV was found to be 10.27, for BF was 10.27, and for k-trans was 2.75. The negative likelihood ratio (LR-) for BV was found to be 0.07, for BF was 0.07, and for k-trans was 0.

**Table 2:** Diagnostic accuracy of perfusion parameters in differentiating between benign and malignant lesions. BV: blood volume; BF: blood flow; AUC: area under the curve; PPV: positive predictive value; NPV: negative predictive value; LR+: positive likelihood ratio; LR-: negative likelihood ratio.

	BV	BF	k-trans
AUC	0.92	0.89	0.86
95% CI	0.83-1.0	0.77-1.0	0.72-1.0
Youden index	0.842	0.84	0.636
Cut-off value	7.56	50.86	5.39
Sensitivity	93.33% (77.93-99.18)	93.33% (77.93-99.18)	100% (88.43-100)
Specificity	90.91% (58.72-99.77)	90.91% (58.72-99.77)	63.64% (30.79-89.07)
PPV	96.55% (81.17-99.45)	96.55% (81.17-99.45)	88.24% (77.44-94.25)
NPV	83.33% (56.4-95.08)	83.33% (56.4-95.08)	100%
Accuracy	92.68% (80.08-98.46)	92.68% (80.08-98.46)	90.24% (76.87-97.28)
LR+	10.27 (1.58-66.7)	10.27 (1.58-66.7)	2.75 (1.26-6.01)
LR-	0.07 (0.02-0.28)	0.07 (0.02-0.28)	0

## Discussion

In our study, the median value of BV for malignant lesions was 11.35 ml/100 gm, and for benign lesions was 5.5 ml/100 gm. The threshold value for BF was 7.56 ml/100 gm. In a study by Shan et al. [9], the median value of BV for malignant lesions was 4.28 ml/100 gm, and for benign lesions was 2.08 ml/100 gm, and the threshold value was 2.5 ml/100 gm. Shan et al. [9] showed BV to have an accuracy of 85%, sensitivity of 98%, and specificity of 64%. In a study by Sitartchouk et al. [6], for differentiating between benign and malignant lesions, BF had an accuracy of 82.1%, sensitivity of 80.4%, and specificity of 100%. In a study by Li et al. [8], BV showed an accuracy of 92.6%, sensitivity of 93.5%, and specificity of 90.9%. Our study has also shown comparable results, with BV showing an accuracy of 92.68%, sensitivity of 93.3%, and specificity of 90.01%.

In our study, the median BF for benign lesions was 45.5 mL/100 g/min, and for malignant lesions was 61.77 mL/100 g/min. The threshold value for BF was 50.86 mL/100 g/min. In the study by Shan et al. [9], the median value for BF in malignant lesions was 93.9 mL/100 g/min, and for benign lesions was 41.61 mL/100 g/min, and the threshold value was 55 mL/100 g/min. Shan et al. [9] showed BF to have an accuracy of 80%, sensitivity of 91%, and specificity of 64%. Sitartchouk et al. [6] showed that for differentiating between benign and malignant lesions, BF had an accuracy of 82.1%, sensitivity of 80.4%, and specificity of 100%. Our study has also shown comparable results, with BF showing an accuracy of 92.68%, sensitivity of 93.3%, and specificity of 90.01%.

In our study, the median value of k-trans in benign lesions was 4.2 mL/100 g/min, and in malignant lesions was 12.2 mL/100 g/min. In the study by Sitartchouk et al. [6], k-trans was 13.3 ± 1.2 ml/100 g/min in malignant lesions and 3.9 ± 0.8 ml/100 g/min in benign lesions. It had an accuracy of 94.1%, sensitivity of 95.7%, and specificity of 80%. Our study has shown k-trans to have an accuracy of 90.24%, sensitivity of 100%, and specificity of 63.64% (Table 3).

**Table 3:** Perfusion parameters of malignant and benign pulmonary lesions in different studies. BV: blood volume; BF: blood flow.

Study		BV (ml/100 gm)	BF (ml/100 gm/min)	k-trans (ml/100 gm/min)
Shan et al. [9]	Malignant	4.28	93.9	
	Benign	2.08	41.61	
Li et al. [8]	Malignant	33.1	61.5	
	Benign	3.4	13.1	
Sitartchouk et al. [6]	Malignant	9.3	111.3	13.1
	Benign	4.1	39.1	3.9
Our study	Malignant	11.3	61.7	12.05
	Benign	5.5	45.5	4.2

Our study has shown k-trans to have the highest sensitivity while BV and BF had a comparable specificity and accuracy in differentiating between benign and malignant lesions.

Our study is limited by the low number of benign nodules (11 out of 41), which has a potential impact on statistical analysis. However, even though the small number of benign nodules affects the reliability of accuracy estimations from the ROC curves, ROC analysis of our data demonstrated high accuracy, sensitivity, and specificity for BV, BF, and k-trans (area under the curve = 0.92, 0.89, and 0.86, respectively).

## Conclusions

Our study has shown that BV, BF, and k-trans can be used to differentiate between benign and malignant focal lung parenchymal lesions with k-trans being the most sensitive parameter while BV and BF have greater accuracy and specificity. As contrast-enhanced CT is the imaging technique of choice for lung cancer, CT perfusion can be done in the patients referred for the scan with the use of contrast. CT perfusion can help us differentiate between benign and malignant lesions and can guide us on which lesions to biopsy, thus reducing biopsy-induced complications and the time interval between diagnosis and initiation of therapy.
